# Measures and time points relevant for post-surgical follow-up in patients with inflammatory arthritis: a pilot study

**DOI:** 10.1186/1471-2474-10-50

**Published:** 2009-05-14

**Authors:** Gunnel Sandqvist, Pia Malcus Johnsson, Anna-Lena Sturesson, Magnus Tägil, Pierre Geborek

**Affiliations:** 1 Department of Rheumatology, Lund University Hospital, SE-221 85, Lund, Sweden; 2 Hand Unit, Department of Orthopedics, Lund University Hospital, Lund, Sweden

## Abstract

**Background:**

Rheumatic diseases commonly affect joints and other structures in the hand. Surgery is a traditional way to treat hand problems in inflammatory rheumatic diseases with the purposes of pain relief, restore function and prevent progression. There are numerous measures to choose from, and a combination of outcome measures is recommended. This study evaluated if instruments commonly used in rheumatologic clinical practice are suitable to measure outcome of hand surgery and to identify time points relevant for follow-up.

**Methods:**

Thirty-one patients (median age 56 years, median disease duration 15 years) with inflammatory rheumatic disease and need for post-surgical occupational therapy intervention formed this pilot study group.

Hand function was assessed regarding grip strength (Grippit), pain (VAS), range of motion (ROM) (Signals of Functional Impairment (SOFI)) and grip ability (Grip Ability Test (GAT)). Activities of daily life (ADL) were assessed by means of Disabilities of the Arm, Shoulder and Hand Outcome (DASH) and Canadian Occupational Performance Measure (COPM). The instruments were evaluated by responsiveness and feasibility; follow-up points were 0, 3, 6 and 12 months.

**Results:**

All instruments showed significant change at one or more follow-up points. Satisfaction with activities (COPM) showed the best responsiveness (SMR>0.8), while ROM measured with SOFI had low responsiveness at most follow-up time points. The responsiveness of the instruments was stable between 6 and 12 month follow-up which imply that 6 month is an appropriate time for evaluating short-term effect of hand surgery in rheumatic diseases.

**Conclusion:**

We suggest a core set of instruments measuring pain, grip strength, grip ability, perceived symptoms and self-defined daily activities. This study has shown that VAS pain, the Grippit instrument, GAT, DASH symptom scale and COPM are suitable outcome instruments for hand surgery, while SOFI may be a more insensitive test. However, the feasibility of this protocol in clinical practice awaits prospective studies.

## Background

Rheumatic diseases commonly affect joints and other structures in the hand. Reduced joint mobility, loss of grip strength, pain and symptoms from nerves and tendons contribute to disturbed hand function and difficulties to perform activities of daily life [1].

Surgery is a traditional way to treat hand problems in inflammatory rheumatic diseases with the purposes of pain relief, restore function and prevent progression [2]. Hand surgery has mainly been evaluated by objective measures such as range of motion (ROM), prosthesis survival and grip strength but also subjectively such as pain, function and disability [3-8]. Disability is according to the International Classification of Functioning, Disability and Health (ICF) an umbrella term [9] that includes health related components classified in body functions and structures, as well as activity and participation. Hand function assessments on the body functions and structures level include measures of ROM, grip ability, grip strength, pain and sensation, while *self-reported questionnaires, interviews and observations *are used to highlight activity limitations.

There are numerous instruments to choose from and no evaluation covers all aspects of hand function [10]. Nordenskiöld [11] proposes that a combination of outcome measures are used and should include instruments to evaluate grip strength, pain and the persons own perceived ability to perform daily activities. Studies including the patient perspective are however scarce [12-15], even though this topic was highlighted on the Outcome Measures in Rheumatology Clinical Trials (OMERACT) conferences in 2000 [16]. Furthermore, the OMERACT filter for outcome measures recommends that instruments should be evaluated according to the criteria of truth, discrimination, and feasibility [17]. The truth criteria include validity, discrimination captures the issues of reliability and sensitivity to change and feasibility addresses the practical reality of the use of the instrument. The aim of the present study was to evaluate if instruments commonly used in rheumatologic clinical practice are acceptable as outcome measures of hand surgery, and to identify time points relevant for post-surgical follow-up.

## Methods

### Study design

Prospective observational study of consecutive patients hospitalised for hand surgical interventions. Longitudinal follow up at time points 0, 3, 6 and 12 months. Instruments evaluated by responsiveness and feasibility.

The inherent element of quality control characterizing the pilot study met the legislative documentation required in Sweden; therefore, no formal approval from the ethical committee was necessary.

### Setting and patients

Inclusion criteria were patients with rheumatoid arthritis (RA) or psoriatic arthritis undergoing hand surgery and a need for post-surgical occupational therapy intervention. The study was performed at one university rheumatologic department in cooperation with hand surgeons from the orthopaedic department. Three experienced occupational therapists were involved in the study.

### Instruments

The instruments selected should include assessments at the level of body functions and activity. Furthermore, the instruments should be reliable and valid, in a Swedish version, and easy to perform for both patient and investigator.

The Health Assessment Questionnaire (HAQ) [18] was used at baseline as an overall measure of functional ability. An overview of the instruments are shown in additional file 1: Table S1.

#### Body function level

Grip strength was measured by the Grippit instrument, which register both maximum force (peak) and average force in Newton (N) over 10 s. The average value over 10 seconds was used in the current analysis. Both treated and non-treated hands were measured. The instrument has good validity in relation to the Jamar instrument [19] and high test-retest scores [20].

Perceived pain during the day in the hands, treated and non-treated separately, were evaluated on a visual analogue scale, VAS ranging from 0 mm = no pain to 100 mm = maximal pain.

Signals of Functional Impairment (SOFI) is a performance test measuring ROM in the hand, arm and lower extremities. In this study we used the hand assessment which consists of 4 items measuring opening grip of the hand, finger-flexion, pincer grip and thumb opposition. Each item has 3 well defined scale steps translated into no impairment (= 0), slight to moderate impairment (= 1), severe impairment (= 2) yielding a total range of SOFI hand score for right and left hand of 0–16. SOFI has acceptable validity and reliability in patients with RA [21].

Grip ability was measured with the Grip Ability Test (GAT) consisting of three items; 1. put a flexi grip stocking over the non-dominant hand, 2. put a paper clip on an envelope and 3. pour water from a jug to a mug. The score (10–276) is based on the time consumption of the 3 items. The mean value (range) for normal hand function is 16.5 s (11.0 – 20.0). GAT has been developed especially for patients with RA with the purpose of measuring the outcome of therapeutic programs. The instrument has high reliability and internal consistency in patients with RA [22].

#### Activity and participation level

Patients were asked to complete the Disabilities of the Arm, Shoulder and Hand Outcome (DASH) form [23]. DASH is a self-administered questionnaire with 30 items on disability and symptoms during the preceding week. Response options range from 1 to 5 (1 = no difficulty, 2 = mild difficulty, 3 = moderate difficulty, 4 = severe difficulty, 5 = unable to do). There are four additional questions about work activities and four about leisure activities. The total DASH produces scores between 0 and 100 for each module. A high DASH score indicates severe disability. In the original, DASH values for items of disability and function produce a single score. In this study we adapted the algorithm to obtain two sub-scores; DASH activity including 21 items (item 1–21), and DASH symptoms including 9 items (item 22–30) [23]. Both scores ranging between 0 and 100. The DASH symptom scale will in this study be subordinated to the body function level. The Swedish version of the DASH questionnaire is reliable and valid [24].

The performance of daily activities and satisfaction with the performance, as perceived by the patient, were assessed with Canadian Occupational Performance Measure (COPM) [25]. COPM is a semi-structured interview used to assess occupational performance and identifies activities in self-care, productivity and leisure. Patients define important activities and rate them according to ability to perform the activity and satisfaction with performance. A ten-point Likert scale is used for ratings, ranged from 1("not able to perform" and "not at all satisfied") to 10 ("perform extremely well" and "extremely satisfied"). Scores of performance and satisfaction are summed separately and divided with the number of activities given by each patient. COPM has good construct validity [26] and concurrent validity against HAQ [27]. In this study the Swedish version is used [28]. In order to optimise the relevance of the instrument in this study the patients were asked to identify activities involving the hand eligible for surgery.

### Data analyses

Wilcoxon signed-rank test was used to test for differences at baseline between treated and non-treated hands, and for testing change over time in both hands. Responsiveness of the different instruments was evaluated by standardized mean response (SMR) e.g. mean change/standard deviation of change. SMR was classified as medium (>0.5) or large (>0.8) [29]. Correlations were evaluated by Spearman's rank correlation coefficient. The software used was SPSS package version 11.

## Results

### Sample

Altogether 37 patients fulfilled the inclusion criteria. Six patients were excluded because of difficulties to contribute in the test procedure of the COPM. These patients had difficulties in selecting goals of performance of daily activities and problems with the scoring procedure. The characteristics of the 31 final patients are given in Table 1. Four of the 31 patients declined to complete the 12 month follow up due to travel inconvenience.

**Table 1:** Clinical characteristics and type of surgery in the patients

	Median (range)
No. of male/female	7/24
Age (years)	56 (28 – 81)
Disease duration (years)	15 (1–32)
HAQ	1.25 (0.38 – 2.38)
*Diagnoses (No.)*	
RA	29
Psoriatic arthritis	2
*Medical treatment (No.)*	
DMARD and TNF-blockers	5
DMARD	21
NSAID/none	5
*Type of surgery (No)*	
Joint surgery	
MCP implant arthroplasty	6
Arthrodesis in the wrist	4
Arthrodesis in fingers	1
Soft tissue surgery	
Tenosynovectomy	6
Carpal tunnel release	1
Centralization of extensor tendons	3
Two procedures in one hand^x^	10

^x ^mostly a combination of arthrodesis in the wrist and arthroplasty in the CMC 1 joint

Disease modifying medical treatment remained stable during follow up except for two patients initiating biologic treatment during follow up period. One patient started etanercept 7 months after surgery and one patient was treated with anakinra between 1 and 3 months after surgery.

Ten patients had surgical procedures performed on soft tissues such as carpal tunnel release, tenosynovectomy and recentralisation of extensor tendons, while 11 patients had bone and joint interventions, arthrodesis or implants performed. In 10 patients there were two or more procedures performed at the same surgical occasion (Table 1). The most frequent combination was arthrodesis in the wrist and arthroplasty in the CMC 1 joint.

The dominant hand was treated in 77% of the patients.

### Occupational therapy

Occupational therapy was the only rehabilitation service provided for all patients included. In mean 4 days of in-hospital treatment was provided, thereafter the rehabilitation was conducted as out-patient visits by the same therapist. The number of treatments per patient was in median 9 (3–42), and each individual session lasted between 15 minutes to 2 hours. Twenty-four patients received one or more splints, either prefabricated or individually made. All patients received instructions regarding hand exercise and most patients received advice aimed to facilitate activities of daily living such as ergonomic instructions and assistive devices.

### Outcome on body function level – Hand function

#### Treated and non-treated hands at baseline

Baseline function in the treated hand was generally more impaired compared to the non-treated hand. Grip strength was in median 58 N (2 – 254) in the treated hand compared to 64 N (7 – 378) in the non treated hand (p = 0.08). VAS hand pain was in median 50 (4 – 100) in the treated hand compared to 20 (0 – 70) in the non-treated hand (p < 0.01), and ROM measured with SOFI was in median 3 points (0 – 7) in the treated hand compared to 2 points (0 – 5) in the non treated hand (p < 0.05).

#### Change during the follow-up period

The results of the hand instruments at baseline and 3, 6 and 12 months follow-up in the treated hand are given in Table 2. Measurement of pain, grip strength, grip ability and DASH symptoms showed the greatest change during the follow-up period. Pain showed the best improvement at the 3- and 6-month follow-up, while grip strength showed the greatest improvement at the 12-month follow-up. The change in DASH symptom scale was significant during all follow-up points. The non-treated hand did not improve significantly in any of variables during the follow-up period (data not shown).

**Table 2:** Performance of hand function, symptoms and activities, at baseline and at 3, 6 and 12 month follow-up

	Baseline Median (range) (n = 31)	3-month follow-up Median (range) (n = 28)	6-month follow-up Median (range) (n = 31)	12-month follow-upMedian (range)(n = 27)
Body function				
Grippit (N) (average value over 10 s)	57 (2–254)	54 (0 – 317)	70 (0 – 319)**	88 (8 – 354)***
VAS general pain (0–100)	50 (4–100)	14 (0 – 65)***	9 (0 – 75)***	20 (0 – 90)*
SOFI	3 (0–7)	3 (0–5)	3 (0 – 6)*	3 (0 – 6)
GAT	31 (13–183)	22 (9.2 – 148.6)*	22 (9.2 – 159.8)***	23.8 (13.6 – 147.2)**
DASH, symptoms (0 – 100)	50 (25 – 83.3)	30.6 (11.1 – 66.7)***	36.1 (0 – 77.8)***	27.8 (0 – 61.1)***
Activity and participation				
DASH, activities (0 – 100)	51.2 (11.9 – 83.3)	42.9 (11.9 – 82.1)**	42.3 (3.6 – 82.1)	44 (4.8 – 78.6)**
COPM (0 – 10)				
Performance of activities	3.8 (1 – 7.3)	5.8 (1 – 9.4)**	6 (2 – 10)***	5.7 (1 – 9.2)***
Satisfaction with performance	2 (1 – 6.7)	5.3 (1 – 9.8)***	5.5 (1.3 – 10)***	5 (1 – 10)***

* = p < 0.05, ** = p < 0.01, *** = p < 0.001

Bold figures represent significant differences between baseline and the different follow-up points

### Outcome on the Activity and Participation level – Performance of daily activities

The median score for the DASH activity was 51 points at baseline. The score changed significantly at the 3- and 12- month, but not at 6-month, follow-up (Table 2).

In the COPM, the patients identified in median 3 (1–5) activities, all related to function of the hand eligible for surgery. The majority of activities, 49%, were within the productivity area which includes work and household activities, 30% were within self-care, and 21% were within leisure activities. The most frequently reported activity problems were; brushing teeth, do and undo buttons, peel potatoes, cut bread and meat, and needlework. But patients reported activity problems of all kinds, e.g. leash the dog, shake hands, fuel the car, lift up a grandchild, gardening and take photographs.

Performance of daily activities and satisfaction with performance improved significantly at all follow-up points (Table 2).

### Correlations between instruments

At baseline there was a significant correlation between all of the objective instruments of hand function (Grippit, SOFI and GAT) (p < 0.01). The subjective measures (VAS hand pain and DASH symptoms) did not correlate significantly with the objective instruments except a significant correlation between DASH symptoms and grip strength (p < 0.001) at baseline. The correlation between delta values (baseline values – follow-up values) was however significant concerning VAS hand pain and grip strength at 12 month follow-up (p < 0.05). Furthermore, we found at baseline a significant correlation between the hand function instruments (Grippit, SOFI and GAT) and performance of activities (COPM and DASH activity) (p < 0.05) and between COPM performance and DASH activity (p < 0.01). The delta values of COPM performance and DASH activity was also significant correlated at 6 month follow up (p < 0.05) No other correlations between the instruments was found.

### Responsiveness to change of the instruments

**Figure 1:**
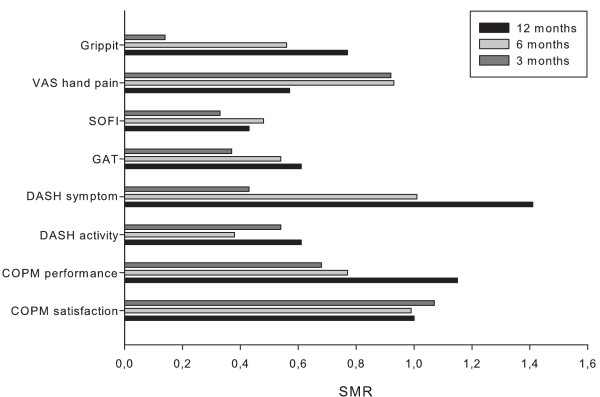
**SMR for the different instruments at follow up 3, 6, and 12 month**.

Responsiveness for the different instruments at follow up 3, 6, and 12 months are illustrated in figure 1. Overall COPM satisfaction showed the best responsiveness, with SMR consistently above 0.8, but also COPM performance had SMR between moderate and large. SOFI had low responsiveness at all follow-up time points with SMR between small (0.2) and moderate (0.5). The Grippit instrument obtained a very low responsiveness at the 3-month follow-up, but this improved considerably at the 12-month follow-up. Moreover, a higher SMR at the 12-month follow-up time point was a common feature for most of the instruments.

## Discussion

This pilot study indicates that a core set of measures of pain, grip strength, grip ability, perceived symptoms and self-defined daily activities gives a broad evaluation of hand surgery. Among the instruments used in this study the COPM was one of the best integrated as it comprises both functional performance and patient satisfaction components. COPM was also highly sensitive to change at all follow-up points, and was the only instrument with a client-centred perspective. The COPM has also shown sensitivity for change in patients with silastic metacarpophalangeal joint protheses [13].

DASH symptom scale and VAS hand pain were two rapidly performed subjective instruments of function. Both tests were moderate to highly sensitive to change during the follow-up period. Pain is also one of the most commonly used outcome measures of hand surgery, and changes in hand pain has previously been shown to be related to patient satisfaction with the outcome of the surgical intervention [4,13].

One aim of the present study was to evaluate if instruments commonly used within rheumatologic care could be included in a core set of outcome measures for hand surgery irrespective of type of surgical procedure. Several instruments in our study have actually shown applicability also in studies evaluating diverse specific surgical procedures such as arthroplasties in metacarpophalangeal joint and proximal interphalangeal joint, and carpal tunnel syndrome [5,12,13]. Even if different surgical procedures are likely to differ in outcome on hand pain and function [4], it is not obvious that there is a need for specifically developed instruments for each surgical procedure. There are several hand function instruments that could be taken into account in the evaluation as outcome measures of hand surgery. After searching the literature, we found that tools assessing pain, grip strength and functional abilities were the most common. In order to fulfil the OMERACT criteria of truth we only used instruments reliable and valid in a Swedish version. A validated native language version for performance tests may not be a prerequisite, but since the validated and currently used performance test were quick and easy to perform, we refrained from introducing new instruments such as the Sequential occupational dexterity assessment (SODA) [30], and the Arthritis hand function test [31]. Both instruments are developed especially for the rheumatic hand and measures bimanual functional abilities, but neither of them has been tested in a Swedish version. The SODA has shown small clinical effect (SMR 0.38) 6-months and 12-month after surgical interventions [4]. Despite this rather small clinical effect Lankveld et al. found that observed dexterity, using instruments like SODA is more sensitive to change than self-report dexterity [4].

In the evaluations of interventions in rheumatic diseases, there is an increase in the use of standardized self-report instruments, both concerning hand function and performance of daily activities. The HAQ is one of the most establish self-reported instrument concerning performance of daily activities in rheumatic diseases. However, HAQ measures several body functions outside the hand, and in this study we therefore used HAQ only as a baseline patient characteristic. Instead we used the DASH instrument with specific hand related activities as an outcome measure. However, showing the patients a list of predefined available items may facilitate comparisons, but disability is the person's individual perception of functioning and not simply a result of being able or unable to do certain activities. COPM is a generic and individualized instrument designed to detect changes in patients' self perception of performance of daily activities and satisfaction with performance over time. This individual design of the instrument ensures Beaton et al [32] recommendations that outcome measures should allow patients to generate their own item content to express how the disorder affects them and could be a reason for the high responsiveness found in the present study. We asked the patient to identify activities involving the hand eligible for hand surgery in order to optimize the relevance of the instrument. Problem that was mentioned were e.g. do and undo buttons and peel potatoes. However, there were some patients (6/37) being excluded because of difficulties in contributing to the test situation, the main problem was with the scoring procedure. This problem has also been reported by others [33] and illustrates some limitation of the COPM instrument. Furthermore, COPM require a personal contact in the pre- and post-surgical process, which also applies to the hand function tests. This may affect the feasibility of the instruments, but in the care of the rheumatic patient undergoing surgery the need of personal contact poses no problem, since these patients often are frequent visitors to the clinic for a number of reasons.

Several clinical factors can have an impact on the evaluation of responsiveness. Appropriate time over which responsiveness should be evaluated can vary and different instruments may be more responsive at different periods of recovery [7]. The sensitivity to change was rather stable for VAS hand pain but there was a tendency toward a better improvement in the early recovery phase. Most surgery procedures give pain relief early in the recovery and instruments focusing on pain therefore may be more responsive in the early phase, as is the case with distal radius fracture [7]. Range of motion measured with the SOFI instrument obtained the poorest sensitivity to change which is not surprising since SOFI focus on motion of the thumb and finger flexion and few surgical procedures aimed to improve these functions in this study. However, the small sample size in this pilot study yields low precision in some of the SMR estimates, which emphasize the need of studies with larger sample size. Larger sample size would allow studies on subgroups such as different rheumatic diseases, overall functional level, and type of surgical procedure (e.g. soft versus hard tissue surgery).

The optimal follow up time point is depending on several factors such as diagnosis, surgical procedure, and patient specific items such as overall functional level, disease progression, medication, and co-morbidity [13,34]. Therefore, when introducing a standardized protocol or core set for quality control of all kinds of rheumatoid hand surgery, compromises have to be made. We aimed to find one predefined core set and follow up time point to be used in the busy every day clinical practise setting. In the present pilot study we only included patients with inflammatory joint diseases and the medication was stable with few exceptions. With increasing time disease activity and drug treatment will inevitably influence the evaluation of surgical procedures. Since these factors can vary considerably over time the short-term effect is perhaps the most appropriate. However, 3 month follow up is too early to evaluate numerous outcome variables, illustrated by low SMR for several of the instruments tested. Considering the high SMR values of several of the instruments and their stability at 6 and 12-month follow-up, and in view of the dropping of some patients at the 12-month follow-up, we propose that an acceptable time to follow up short-term effect from hand surgery in rheumatic diseases is at 6 months.

## Conclusion

Supported by the large SMR:s values we suggest that a core set of instruments for evaluating outcome of hand surgery in rheumatic diseases should include measurement of pain, grip strength, grip ability, perceived symptoms and self-defined daily activities. This study has shown that VAS pain, the Grippit instrument, GAT, DASH symptom scale and COPM are suitable outcome measures, and presumably reducing to Grippit, COPM and DASH symptom are satisfactory for most situations. Because of the rather poor SMR in SOFI we can not recommend the test as a sensitive outcome measure. However, the feasibility of this protocol in clinical practice awaits prospective studies.

## Competing interests

The authors declare that they have no competing interests.

## Authors' contributions

GS and PMJ collected and analysed the data and is the principle authors of the paper. PG has made substantial contributions in analysis and interpretation of data and in revising the manuscript critically. ALS collected the data and has made analysis and interpretation of data and MT provided a critical review of the manuscript. All authors read and approved the final manuscript.

## Pre-publication history

The pre-publication history for this paper can be accessed here:



## Supplementary Material

Additional File 1 **Table S1**. Characteristics of the different instruments. Click here for file
