# Supplementary material for: Measures and time points relevant for post-surgical follow-up in patients with inflammatory arthritis: a pilot study
Source: BMC Musculoskelet Disord. 2009 May 14;10:50. doi: 10.1186/1471-2474-10-50 (PMC2691396; doi:10.1186/1471-2474-10-50)
Supplement: Additional File 1 — Table S1. Characteristics of the different instruments. [file 1471-2474-10-50-S1.doc]

Table S1. Characteristics of the different instruments

| Instrument | ICF | Measures | Outcome type | Left/right  selective | Approximate time to perform |
| --- | --- | --- | --- | --- | --- |
| Grippit | Body function | Grip strength | Objective in Newton | Yes | 1-5 minutes |
| VAS hand pain | Body function | Pain | Subjective in mm | Yes | <1 minute |
| Signals of functional impairment (SOFI) | Body function | ROM | Objective in points | Yes | 5 minutes |
| Grip ability test (GAT) | Body function | Grip ability | Objective time in seconds | Not applicable | 5 minutes |
| Disabilities of the Arm, Shoulder and Hand Outcome (DASH) | Activity and participation | Standardized activities and symptoms | Subjective in points | Not applicable | 15 minutes |
| Canadian Occupational Performance Measure (COPM) | Activity and participation | Patient specific activities | Subjective in points | Not applicable | 30 minutes initial,10 min at follow up |
